# Assessing the role of programmed cell death signatures and related gene TOP2A in progression and prognostic prediction of clear cell renal cell carcinoma

**DOI:** 10.1186/s12935-024-03346-w

**Published:** 2024-05-10

**Authors:** Qingshui Wang, Jiamin Liu, Ruiqiong Li, Simeng Wang, Yining Xu, Yawen Wang, Hao Zhang, Yingying Zhou, Xiuli Zhang, Xuequn Chen, Wei Zhuang, Yao Lin

**Affiliations:** 1 Innovation and Transformation Center, Second Affiliated Hospital of Fujian University of Traditional Chinese Medical University Medicine, Fujian-Macao Science and Technology Cooperation Base of Traditional Chinese Medicine-Oriented Chronic Disease Prevention and Treatment, Fujian University of Traditional Chinese Medicine, Fuzhou, China; 2 Department of Urology, The Second Affiliated Hospital of Fujian Medical University, Quanzhou, 352000 Fujian Province China

**Keywords:** KIRC, PCD, Immunotherapy, Immune microenvironment, TOP2A

## Abstract

Kidney Clear Cell Carcinoma (KIRC), the predominant form of kidney cancer, exhibits a diverse therapeutic response to Immune Checkpoint Inhibitors (ICIs), highlighting the need for predictive models of ICI efficacy. Our study has constructed a prognostic model based on 13 types of Programmed Cell Death (PCD), which are intertwined with tumor progression and the immune microenvironment. Validated by analyses of comprehensive datasets, this model identifies seven key PCD genes that delineate two subtypes with distinct immune profiles and sensitivities to anti-PD-1 therapy. The high-PCD group demonstrates a more immune-suppressive environment, while the low-PCD group shows better responses to PD-1 treatment. In particular, TOP2A emerged as crucial, with its inhibition markedly reducing KIRC cell growth and mobility. These findings underscore the relevance of PCDs in predicting KIRC outcomes and immunotherapy response, with implications for enhancing clinical decision-making.

**Supplementary Information:**

The online version contains supplementary material available at 10.1186/s12935-024-03346-w.

## Introduction

Kidney Clear Cell Carcinoma (KIRC), also known as Renal Cell Carcinoma (RCC), is the most common primary kidney malignancy in adults. It embodies approximately 75% of all kidney cancers and proves more lethal than its non-clear cell counterparts [1, 2]. The heightened mortality rate of KIRC can be attributed to its propensity for metastasis and resistance to conventional chemotherapy or radiation treatments [3].

Therapeutic and prognostic approaches to kidney cancer have been historically challenging due to the disease’s heterogenous nature and complexity [4]. In the last decade, immune checkpoint therapy, notably the PD-1 pathway blockade, has precipitated groundbreaking advances in cancer treatment. However, responses to immunotherapies, such as PD-1 blockade, present considerable variation between patients, with many exhibiting primary resistance or susceptibility to side effects. The need for novel prognostic biomarkers and therapeutic strategies for KIRC is paramount to enhance survival rates and health outcomes [5].

Understanding the mechanisms of Programmed Cell Death (PCD) unlocks significant potential in developing novel cancer therapies [6]. Various forms of PCD exist, such as apoptosis, endolytic cell death, necroptosis, ferroptosis, reticulocyte death, autophagy-dependent cell death, pyroptosis, parthanatos, cuproptosis, lysosome-dependent cell death, oxidative death, anoikis and alkaline death. Apoptosis is a tightly regulated form of cell death that plays a critical role in numerous homeostatic and pathological processes [7]. Necroptosis is another regulated type of cellular demise that does not rely on the activation pathways of the cysteine family proteases [8]. Ferroptosis is yet another form of controlled cell death, primarily characterized by an overload of iron and the accumulation of lipid peroxides that are dependent on reactive oxygen species (ROS) [9]. Distinguished by plasma membrane pores reliant on caspase-1, pyroptosis leads to the release of pro-inflammatory cytokines and cell lysis [10]. Netotic cell death is shaped by ROS production mediated by NADPH oxidase and histone glutamylation [11]. A process termed Entotic cell death entails one cell enveloping and killing another [12]. Lysosome-dependent cell death is a form of PCD mediated by hydrolases such as cathepsin or iron discharge through lysosomal membrane permeabilization (LMP) and is identified by lysosomal rupture [13]. Parthanatos, also known as PARP-1-dependent cell death, is an innovative form of PCD premised on DNA damage and the activation of PARP-1 [14]. The autophagy-dependent cell death is characterized by cytoplasmic vacuolization, autophagosome formation, and the removal of materials via lysosomes [15]. Oxeiptosis, a new form of PCD, is triggered by oxygen radicals, independent of caspases, and propelled by the KEAP1-PGAM5-AIFM1 pathway [16]. Alkaliptosis, another novel form of PCD, is driven by the internal alkalinization of cells [17]. Cuproptosis is a copper-dependent, controlled form of cell death [18]. Anoikis refers to a process in which normal adherent cells undergo cell death due to a lack of attachment, colloquially known as ‘homelessness’, if they remain suspended for an extended period [19]. Each determining the immunological response to cell death, hence rendering specific cell deaths as either “immunogenic” or “non-immunogenic”. Decoding the mechanisms and implications of diverse cell death modalities could provide substantial insights into cancer immunology, immunotherapy, and drug development.

This study rigorously examined the differential expression of thirteen types of PCD genes in KIRC. We also pioneered a prognostic model for KIRC patients grounded on PCDs (PCD signature) features and assessed their relevance to KIRC’s clinical features. Our focus extended to the capacity of PCDs to reflect the tumor immune microenvironment and their prospective applicability in predicting prognosis and response to PD-1 blockade in KIRC patients. The findings from our study have the potential to augment the efficacy of immunotherapy and expedite the development of innovative therapeutic strategies for KIRC.

## Methods

### Data collection

PCD genes encompass crucial regulatory elements for the 13 aforementioned PCD modalities. These genes were sourced from GSEA gene sets, KEGG, and articles [8]. Among them, a significant number of genes are associated with each distinct cell death mode. Apoptosis is related to 161 genes, Pyroptosis has 27 associated genes, and Ferroptosis is linked to 64 genes. Autophagy, with a relation to 238 genes, and Necroptosis, with 96 genes. Anoikis and Phagocytosis are associated with 35 and 237 genes, respectively. Moreover, Cuproptosis relates to 14 genes, Parthanatos to 9 genes, Entotic cell death to 15 genes, and Netotic cell death to 8 genes. Alkaliptosis, Oxeiptosis, and lysosome-dependent cell death are related to 7, 5, and 170 genes, respectively (Supplementary Table 1).

### Sample collection and data preprocessing

Data of KIRC patients were downloaded from The Cancer Genome Atlas (TCGA) database. After excluding samples without adequate survival information, a total of 528 KIRC patients were analyzed in this study. Additionally, data of 101 KIRC and 181 KIRC patients were used as external validation cohorts obtained from E-MTAB-1980 [20] and Braun 2020 [21] datasets respectively. GSE14994, GSE36895, GSE40435, GSE46699, GSE53757, and GSE66272 were retrieved from the Gene Expression Omnibus (GEO) database. GSE14994 included 59 KIRC tissue samples and 11 adjacent non-tumor tissues. GSE36895 comprised of 29 KIRC tissue samples and 23 adjacent non-tumor tissues. GSE40435 was composed of 101 KIRC tissue samples and 101 correspondent adjacent non-tumor tissues. Moreover, GSE46699 contained 67 samples of KIRC tissues, along with 63 samples of adjacent non-tumor tissues. Similarly, GSE53757 boasted 72 samples of KIRC tissues and 72 of adjacent non-tumor tissues. Lastly, GSE66272 accommodated 27 samples of KIRC tissues and 27 samples of the adjacent non-tumor tissues. Normalization and preprocessing of microarray data were performed using the Robust Multi-array Average (RMA) method within the Affy package in R.

### Differentially expressed gene analysis

The expression levels of genes related to programmed cell death were analyzed in KIRC and normal tissues using R package “limma”. Based on the criteria of |log2FC| ≥ 0.585 and adjusted p-value < 0.05, differentially expressed genes were then selected for downstream analyses.

### Functional enrichment analysis

To investigate the potential biological processes and pathways that these differentially expressed genes might involve, KEGG and Hallmark pathway enrichment analyses were conducted. Adjusted p-value < 0.05 was used as a cutoff for significant pathways or processes.

### Gene mutation analysis

The GSCA database (https://guolab.wchscu.cn/GSCA/#/) is a cancer genomics portal for gene set cancer analysis. In the study, it was used to analyze the relationship between programmed cell death genes expression and single nucleotide variation (SNV) in KIRC.

### Univariate and multivariate Cox analysis

The univariate Cox regression model was used to evaluate the association between each gene and overall survival to screen the survival-associated genes. The Cox proportional hazards model was constructed based on these survival-associated genes by LASSO regression analysis using R package “glmnet”. Both univariate and multivariate Cox regression analyses were done to explore the prognostic value of genes and clinical features.

### Construction and validation of the prognostic model

The prognostic risk score was calculated for each patient according to the expression level and LASSO coefficient values of the contributing genes of the model. The patients in the TCGA-KIRC cohort were stratified into high- and low-risk groups using the median risk score as the cut-off. The prognostic performance of the model was evaluated by plotting survival curves with the log-rank test and ROC curves. The external validation cohorts were also used to validate the robustness of the prognostic model.

### Subgroup clustering and analysis

Based on the contributing genes of the prognostic model, consensus clustering was performed using the R package “ConsensusClusterPlus” to subdivide the KIRC patients into different clusters [22]. Kaplan-Meier survival analysis was used to assess the survival difference between the different clusters.

### Immune infiltration analysis

To estimate the immune infiltration in KIRC, we employed the ESTIMATE, CIBERSORT [23], and XCELL [24]algorithm. We analyzed the correlation between PCDs and immune cells.

### Nomogram construction and validation

The nomogram was built with the rms package based on multivariate Cox regression analysis results. The confusion matrix and the area under the ROC curve (AUC) were applied to evaluate the predictive efficiency of the nomogram. The nomogram performance was determined by performing a bootstrapping validation with 1000 bootstrap resamples.

### Acquisition of cell lines and culturing and transfection processes

The KIRC cell lines 786-O and ACHN were obtained from the American Type Culture Collection (ATCC, Manassas, VA, USA). The 786-O cultures were nurtured in PRMI 1640, and ACHN cell lines in DMEM (Gibco by Life Technologies, Grand Island, NY, USA), supplemented with 10% FBS (BI, Kibbutz Beit Haemek, Israel) and grown in a humidified incubator at 37°C supplying 5% CO_2_. The shRNA1 and shRNA2 sequences aimed at TOP2A were cloned respectively into pLVX vectors. The sequences included; TOP2A shRNA-1, 5’-GCTCCAAATCAATATGTGATT-3’; TOP2A shRNA-2, 5’GCCTGATTTGTCTAAGTTTAA-3’. Transfection was performed employing the PEI transfection kit (Invitrogen) as per the guidelines provided by the manufacturer.

### Cell counting kit-8 assay

Cell proliferation was assessed employing a Cell Counting Kit-8 (CCK-8) assay. Both the ACHN and 786-O cultures were converted to cell suspensions and densified to 1 × 10^4^ cells/ml. The cell suspensions were added into four 96-well plates (2 × 10^3^ cells well) and incubated at 37 °C with 5% CO_2_. After predefined durations of 24, 48, and 72 h, CCK-8 solution (10 µl) was added an hour before recording the absorbance. The absorbance was subsequently read at 450 nm with a microplate reader.

### Scratch-based assay

ACHN and 786-O cultures post-transfection were inoculated on six-well plates (1 × 10^6^ cells/well). To negate the impacts of cell vitality, cultures were serum-starved. A scratch was introduced in the monolayer, with a 10-µl pipette tip at approximately 90% of cell convergence. PBS was used to wash off detached cells and the wound closure was evaluated with an inverted microscope at 0 and 48 h. ImageJ software was employed to compute the migration area.

### Transwell analysis

Cell invasion was examined using polycarbonate membrane Transwell inserts (Costar; Corning Inc.). Post 48 h of transfection, cells were introduced to the upper chamber (2 × 10^4^ cells/well) along with 200 µl serum-free medium. The upper chamber was subsequently incubated for 24 h in a 24-well plate chamber supplied with 200 µl complete medium with 10% FBS.

### RT-PCR analysis

Total RNA was extracted using TRIzol. This RNA was reverse transcribed using an mRNA Reverse Transcription Kit (Takara, Japan). SYBR Green Kit (Vazyme, China) was used for an RT-PCR experiment. The primer sequences were: TOP2A forward primer 5’-TAATCAGGCTCGCTTTATCTT-3’, TOP2A reverse primer 5’-TCCGAATCATATCCCCCTCT-3’; GAPDH forward primer 5’-GGAAGGACTCATGACCACAGTCC-3’; GAPDH reverse primer 5’-TCGCTGTTGAAGTCAGAGGAGACC-3’. GAPDH was used as the control. The 2^−△△CT^ method was used to determine gene expression levels.

### Zebrafish xenograft methodology

Zebrafish were obtained from Fuzhou Bio-Service Biotechnology Co. Ltd. in Fuzhou, China. The ACHN cells underwent labeling with a red-fluorescent lipophilic membrane dye at 5 µM concentration. They were transplanted into zebrafish larvae utilizing a microinjector. Approximately 200 cells were injected into each specimen, with each group consisting of ten zebrafish larvae. To evaluate tumor cell proliferation within the zebrafish, fluorescent images of each group’s ten specimens were captured at two separate time points: 2 h and 48 h after xenotransplantation. Tail fluorescence images were captured at 2 h and again at 24 h post-transplantation to monitor and evaluate distant metastatic activity.

### Statistical analysis

Survival differences between high-risk and low-risk patient groups were tested using a log-rank test. Multiple Cox regression analyses were used to test the independence of the risk score.

## Results

### Variation of programmed cell death genes (PCD) in KIRC patients

**Fig. 1:**
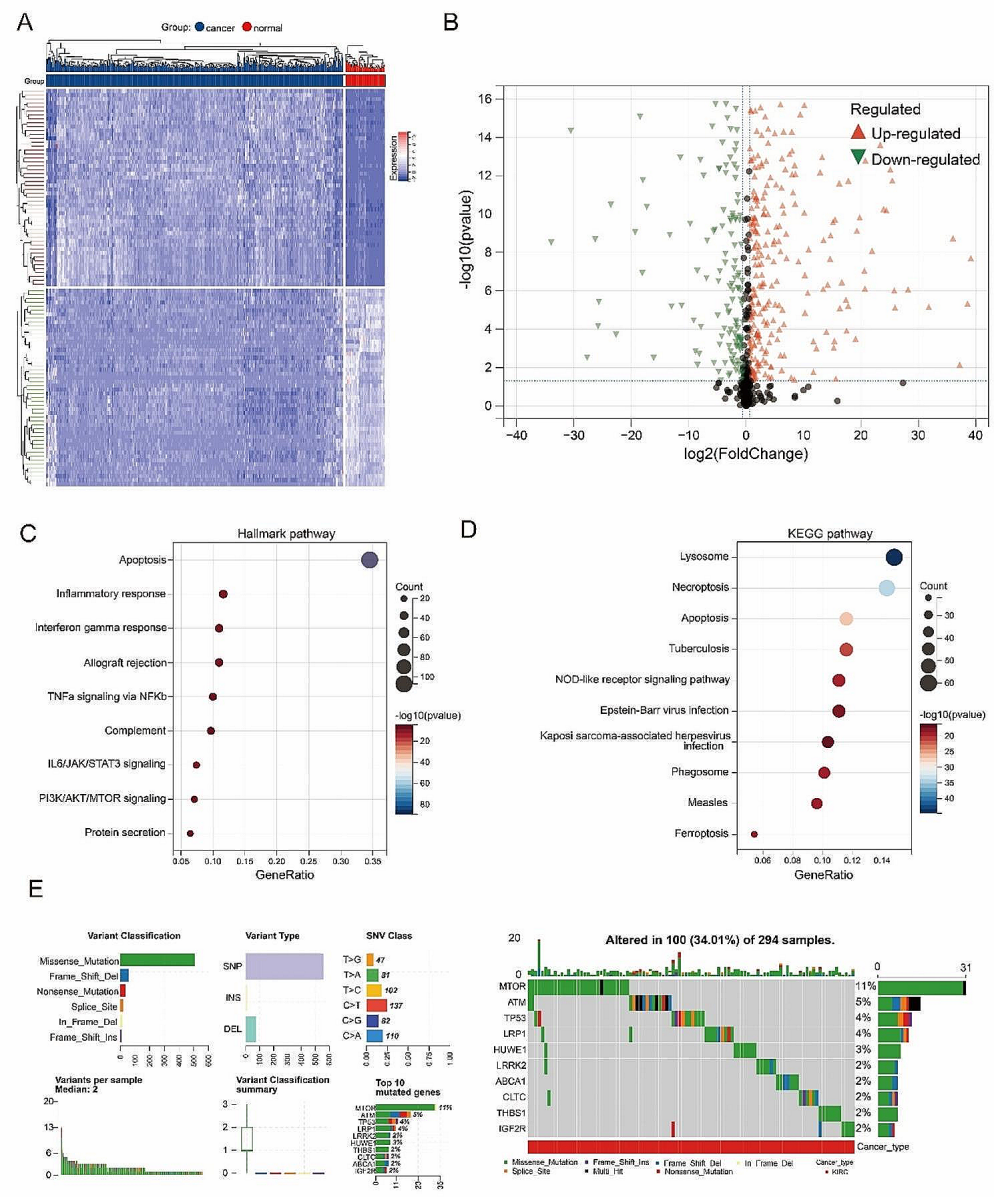
Variant landscape of PCD genes in KIRC patients. (**A**) Heatmap and (**B**) Volcano plot showing differentially expressed PCD genes. Results of (**C**) Hallmark and (**D**) KEGG enrichment analyses for the differentially expressed genes. (**E**) An oncoplot of PCD-related genes in the TCGA cohort

Firstly, we analyzed the expression of thirteen types of programmed cell death genes in kidney clear cell carcinoma. Based on the TCGA-KIRC cohort, we identified 519 differentially expressed genes (Fig. 1A; Supplementary Table 2). Compared to adjacent normal tissue, there were 299 genes with significantly upregulated expression and 220 with significantly downregulated expression in KIRC tissues (Fig. 1B). KEGG and Hallmark pathway enrichment analysis indicated that these differential genes were mainly enriched in pathways such as Apoptosis, Inflammatory response, interferon gamma response, lysosome, necroptosis, and tuberculosis (Fig. 1C&D). Using TCGA-KIRC cohort, we assessed the mutations of programmed cell death genes in KIRC patients. The results showed that 294 KIRC patients had mutations in programmed cell death genes, with MTOR having the highest mutation frequency at 11%, and the mutation frequencies of nine other genes ranged between 2 and 5% (Fig. 1E&F).

### Construction and validation of a prognostic model for KIRC patients based on PCD features

**Fig. 2:**
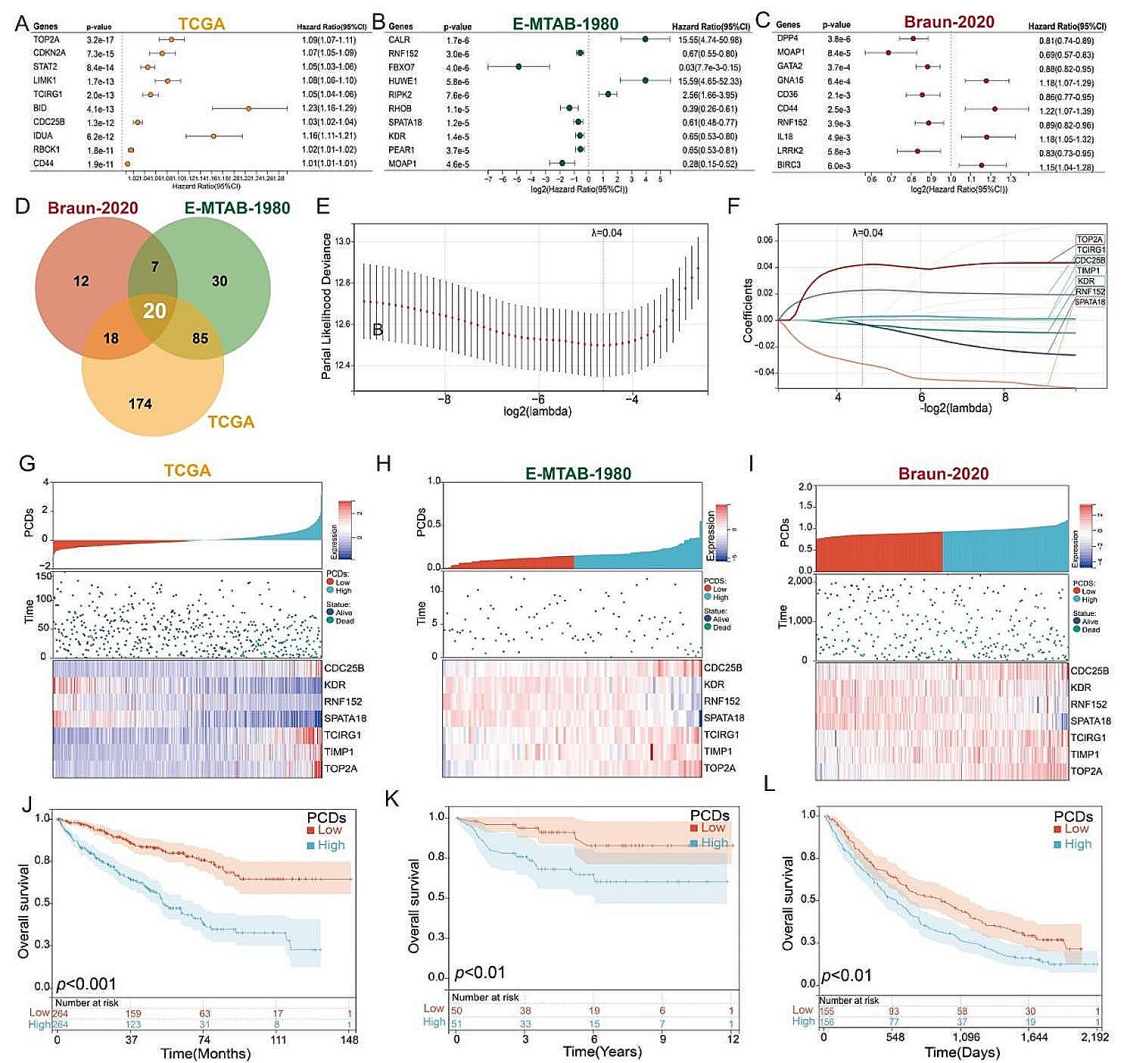
Construction of a prognostic model for KIRC patients based on PCD genes. Univariate survival analysis of differentially expressed PCD genes in (**A**) TCGA, (**B**) E-MTAB-1980, (**C**) Braun-2020 cohorts. (**D**) Venn diagram showing the intersection among the three cohorts. (**E** & **F**) LASSO Cox regression to construct a prognostic model for KIRC patients. (**G**-**I**) Expression levels of PCDs, survival status, and seven genes in the three KIRC cohorts; (J-L) Impact of PCDs on OS of patients in the three KIRC cohorts

Univariate Cox regression analysis was used to screen for survival-related programmed cell death genes. In the TCGA-KIRC cohort, a total of 297 genes were associated with the prognosis of KIRC patients (Fig. 2A; Supplementary Table 3); in the E-MTAB-1980 cohort, there were 142 prognostically relevant genes (Fig. 2B; Supplementary Table 3); and in the Braun-2020 cohort, there were 57 prognostically relevant genes (Fig. 2C; Supplementary Table 3). Venn diagram analysis showed an intersection of 20 genes across these three cohorts (Fig. 2D). Subsequently, we used the TCGA-KIRC cohort as a testing set and utilized LASSO analysis to construct a PCD based on the expression of these 20 genes. The model performed best with 7 genes (Fig. 2E), the regression coefficients of these genes are shown in Fig. 2F. The prognostic model PCDs (programmed cell death genes signature) is formulated as (0.0026 * expression of CDC25B) + (-0.0036 * expression of KDR) + (-0.0029 * expression of RNF152) + (-0.0331 * expression of SPATA18) + (0.0225 * expression of TCIRG1) + (0.0001 * expression of TIMP1) + (0.0418 * expression of TOP2A) (Fig. 2F). Figure 2G demonstrates the PCDs, survival status, and expression levels of the seven programmed cell death genes in the training set of the TCGA-KIRC cohort. Next, we compared the overall survival rate of KIRC patients with different PCDs values. Results indicated that patients with High-PCDs subtype had shorter survival rates than those with Low-PCDs subtype (Fig. 2J; Supplementary Table 4). Then, we used E-MTAB-1980 and Braun-2020 as validation cohorts (Supplementary Table 4). Based on the median PCDs of the validation cohorts, 101 KIRC patients from the E-MTAB-1980 cohort and 311 KIRC patients from the Braun-2020 cohort were divided into two groups. Figure 2H&I show the PCDs, survival status, and expression levels of the seven programmed cell death genes in the validation cohorts of E-MTAB-1980 and Braun-2020, respectively. Kaplan-Meier analysis indicated that patients in the high PCDs group had shorter overall survival and higher mortality rates (Fig. 2K&L).Moreover, the ROC curves for PCDs at 1, 2, and 3 years stood at 0.78, 0.75, and 0.74 respectively in TCGA cohort (Supplementary Fig. 1).

### Unsupervised clustering analysis of PCDs

To explore unidentified KIRC subtypes, we performed unsupervised clustering analysis with seven programmed cell death genes. We discovered that when k = 2, the distinction between subgroups was most significant, suggesting that the 528 KIRC patients could be well categorized into two classes (Supplementary Fig. 2A&B). There was a significant difference in survival time between the two subtypes (Supplementary Fig. 2C). The C2 subtype was associated with a favorable prognosis, while the C1 subtype was linked to a poor prognosis. Similar results were also found in the E-MTAB-1980 cohort (Supplementary Fig. 2E-G) and the Braun-2020 cohort (Supplementary Fig. 2I-K). Moreover, Sankey diagrams illustrated that the majority of the C1 subtype was associated with high PCDs, while the majority of the C2 subtype was associated with low PCDs (Supplementary Fig. 2D&H&L).

### Correlation between PCDs and higher tumor grade and cell cycle progression

**Fig. 3:**
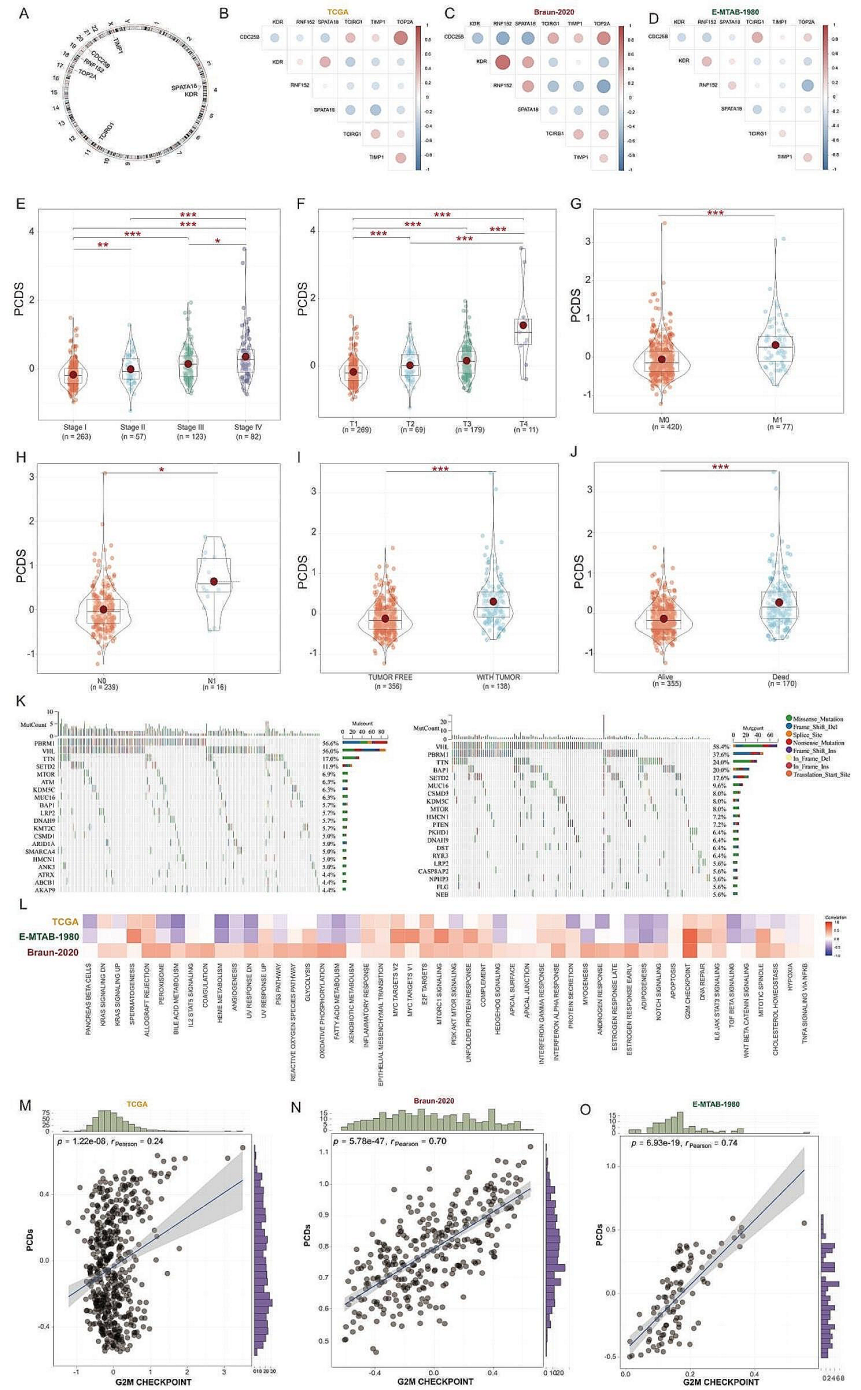
Analysis of the clinical correlation of PCDs with KIRC patients.(**A**)The chromosomal location distribution of 7 genes. the expression correlation analysis of 7 genes in the (**B**) TCGA, (**C**) Braun-2020, and (**D**) E-MTAB-1980 cohorts. (**E**) Differences in PCDs values across different stages. (**F**) Differences in PCDs values across different T classifications. (**G**) Differences in PCDs values across different M classifications. (**H**) Differences in PCDs values across different N classifications; (**I**) Differences in PCDs values between recurrent and non-recurrent patients. (**J**) Differences in PCDs values between living and deceased patients. (**K**) Gene mutation analysis in the High-PCDs and Low-PCDs subgroups. (**L**) Correlation analysis of PCDs with the Hallmark signaling pathways. Correlation analysis of PCDs with the G2M checkpoint in the (**M**) TCGA, (**N**) Braun-2020, and (**O**) E-MTAB-1980 cohorts. *, *p* < 0.05; ***, *p* < 0.001

**Fig. 4:**
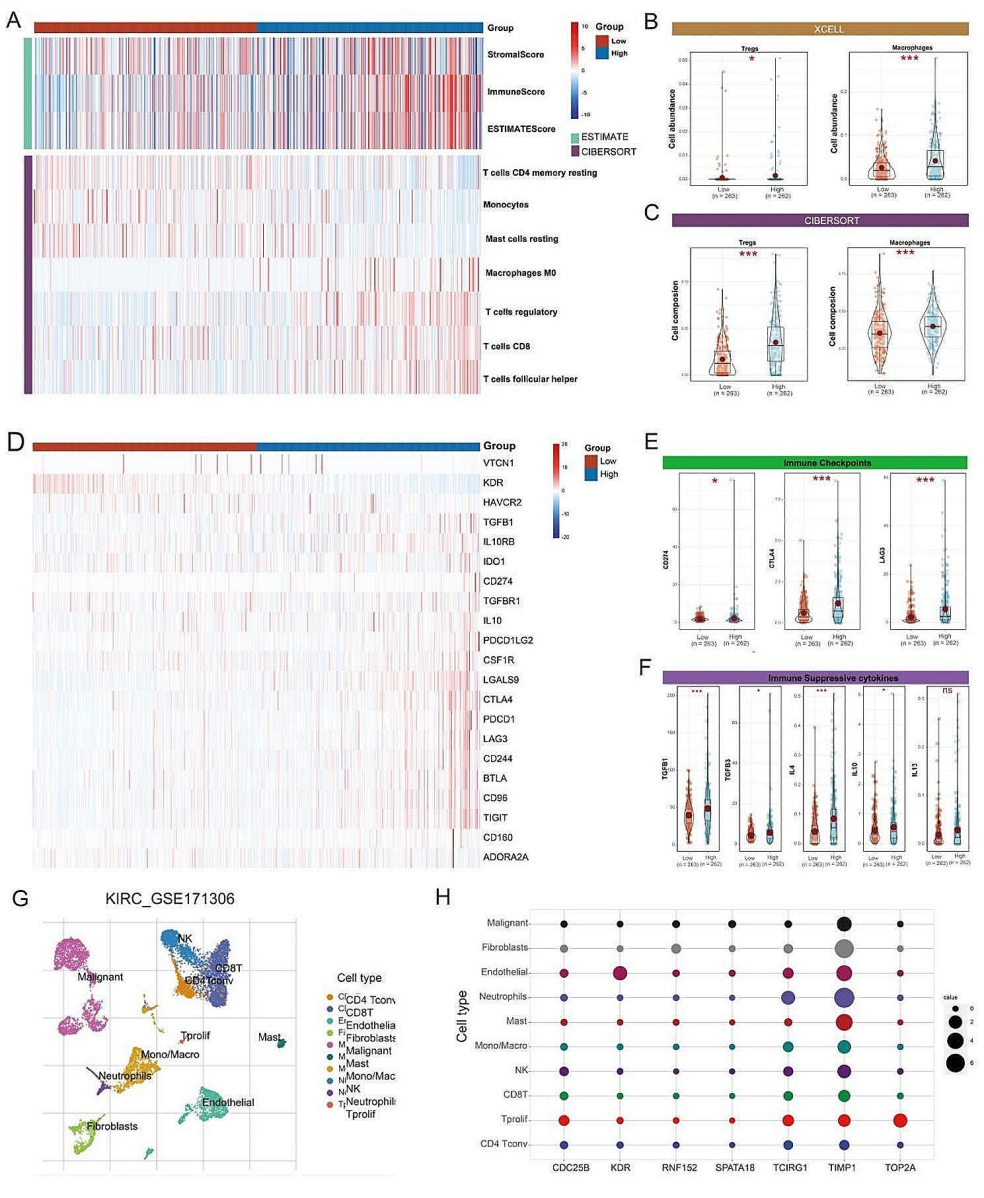
Relationship between PCDs and the Immune Microenvironment. (**A**) Results of estimated scores and differential immune cell infiltration between High-PCDs and Low-PCDs subgroups in TCGA, assessed by CIBERSORT and ESTIMATE. In TCGA, the relative cell abundances of macrophages and Tregs between the two groups are calculated using (**B**) XCELL and (**C**) CIBERSORT. (**D**) Differentially expressed genes profile involved in the negative regulation of the Cancer-Immunity Cycle between High-PCDs and Low-PCDs subgroups. (**E**) Expression of common immune checkpoints between High-PCDs and Low-PCDs subgroups. (**F**) Expression of immunosuppressive cytokines between High-PCDs and Low-PCDs subgroups. (**G**) t-SNE plot visualization of all cell subtypes from KIRC patients in the GSE171306 cohort. (**H**) Bubble plot depicting the expression of model genes across different cell subtypes. ns, *p* > 0.05; *, *p* < 0.05; ***, *p* < 0.001

Subsequently, we evaluated the relevance of PCDs with clinical characteristics of KIRC. Among the seven programmed cell death genes, except for TIMP1 which is located on the X chromosome, the remaining six genes were found on autosomes (Fig. 3A). Figure 3B-D present the expression correlation analysis of these seven genes. Utilizing the TCGA-KIRC cohort, we observed an increasing trend in PCDs values with progression in stage (Fig. 3E). A similar pattern was also discerned across different T stages (Fig. 3F). Significantly higher PCDs values were noted in patients with M1 and N1 compared to those with M0 and N0 (Fig. 4G & H), and the PCDs values were elevated in patients with tumor recurrence compared to those without (Fig. 3I). KIRC patients who were alive exhibited significantly lower PCDs compared to those who had deceased (Fig. 3J). In addition, we investigated the mutational landscape in patients with different PCDs groupings, revealing that VHL, PBRM1, and TTN were among the top three mutated genes in both groups (Fig. 3K). To elucidate the potential mechanisms by which PCDs contribute to KIRC progression, we performed GSVA analysis on data from TCGA-KIRC, E-MTAB-1980, and Braun-2020 cohorts. Figure 4L illustrates the correlations between PCDs and signaling pathways in the three cohorts, with the G2M Checkpoint signaling pathway exhibiting high relevance across all (Fig. 3L). The relationship between PCDs and the G2M Checkpoint signaling pathway across the three cohorts is depicted in Fig. 3M-O. Consequently, KIRC with a High-PCDs subtype is characterized by an upregulated cell cycle process, which may lead to excessive proliferation of tumor cells and adverse outcomes.

### Immune landscape between low-PCDs and high-PCDS subtypes

To assess whether PCDs can reflect the tumor immune microenvironment, we estimated the infiltration of immune cells in KIRC using three independent algorithms: ESTIMATE, CIBERSORT, and XCELL. Immune score results indicated more extensive infiltration of immune cells within the High-PCDs subtype. Moreover, this subtype contained higher levels of immunosuppressive cells (Follicular helper T cells, Regulatory T cells (Tregs), Macrophages) (Fig. 4A). By comparing the results from XCEL and CIBERSORT, both Tregs and Macrophages were significantly upregulated in the High-PCDs subtype (Fig. 4B-C), suggesting the presence of an immunosuppressive phenotype within these tumors.

To further validate the immunosuppressive phenotype, we studied the immune molecules negatively regulating anti-tumor immune responses. Results indicated that genes involved in the negative regulation of the cancer-immunity cycle were generally upregulated in patients with the High-PCDs subtype, suggesting lower activity of the anti-tumor immune process in these patients (Fig. 4D). We compared common immune checkpoints such as PD-L1, CTLA-4, and LAG3 between the two subtypes. Findings showed that PD-L1, CTLA-4, and LAG3 were significantly overexpressed in High-PCDs subtype patients compared to Low-PCDs (Fig. 4E). Chemokines involved in immunosuppression induced by Macrophages and Tregs, such as IL-4, IL-10, TGF-β, were also significantly upregulated in the high-risk group (Fig. 4F). Finally, we explored the expression of model genes in KIRC patients using single-cell RNA transcriptome data (GSE171306) (Fig. 4G). Dot plots demonstrated widespread expression of most model genes across different cell types (Fig. 4H).

### Low-PCDs subtype correlated with enhanced response to anti-PD-1 immunotherapy in patients

**Fig. 5:**
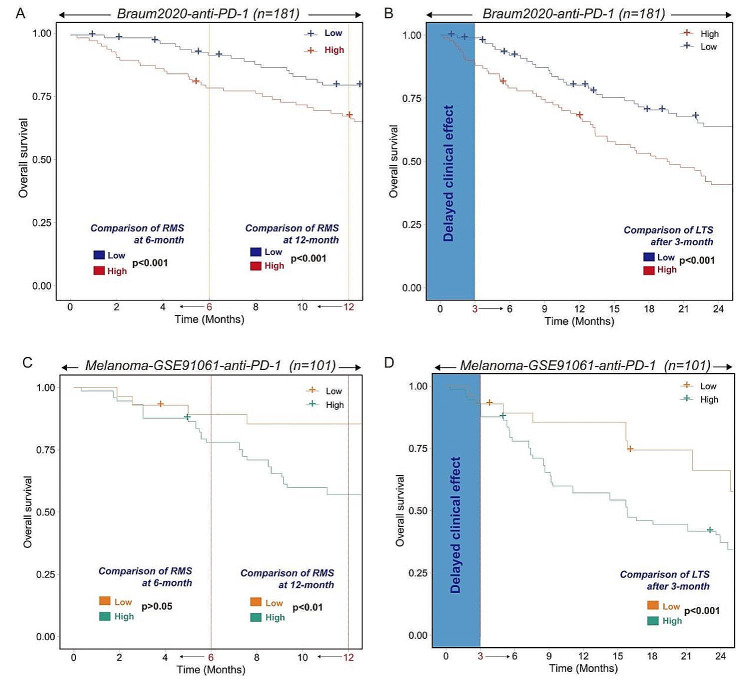
Kaplan-Meier estimator displaying the overall survival curves for High-PCDs and Low-PCDs subgroups, with two non-proportional hazards statistical methods utilized to compare the prognosis of different PCDs. The restricted mean survival (RMS) time difference at six months and one-year post-treatment were compared in (**A**) Braun-2020-anti-PDL1 cohort and (**B**) Melanoma-GSE91061-anti-PDL1 cohort. The first three months following immunotherapy were considered to have a delayed clinical effect, hence the long-term survival post three months of treatment was compared using Chi-square (Qua) approach in (**C**) Braun-2020-anti-PDL1 cohort and (**D**) Melanoma-GSE91061-anti-PDL1 cohort

Given the correlation between PCDs and immune activity in KIRC, we sought to examine if there was an association between PCDs and response to immunotherapy. The Braun 2020 cohort included 181 KIRC patients treated with anti-PD-1 therapy. Survival analysis revealed that the High-PCDs subtype was associated with poorer overall survival (OS) (at 6 months, *p* < 0.001; at 12 months, *p* < 0.001; Fig. 5A). Considering that the clinical effects of immunotherapy can be delayed, we compared the OS rates three months post-treatment and found the High-PCDs subtype similarly correlated with poorer OS (Fig. 5B). On the other hand, a similar analysis in the Melanoma-GSE91061 cohort, consisting of 101 melanoma patients undergoing PD-1 blockade treatment, indicated that the High-PCDs subtype was associated with worse OS in melanoma patients (Fig. 5C-D). These findings suggest that PCDs have the potential to predict the response to immunotherapy across various malignancies, with patients in the Low-PCDs subtype more likely to benefit from immunotherapeutic interventions, and vice versa. PCDs could serve as a promising prognostic marker for the immunotherapeutic response in KIRC patients.

### Nomogram based on PCDs for predicting OS

**Fig. 6:**
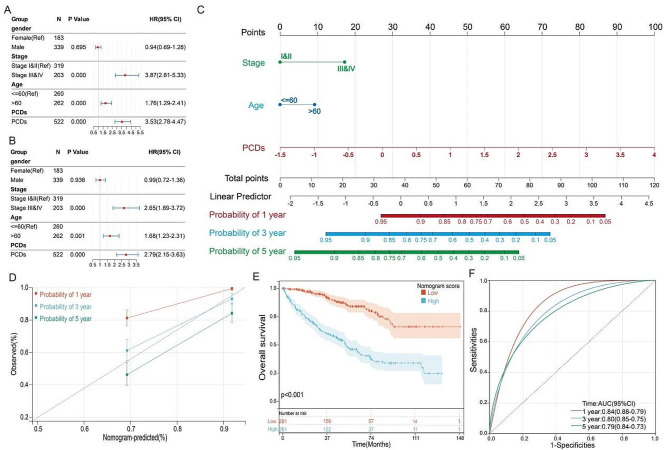
Establishment and assessment of the nomogram survival model. (**A**) Univariate analysis for the clinicopathologic characteristics and PCDs in TCGA cohort. (**B**) Multivariate analysis for the clinicopathologic characteristics and PCDs in TCGA cohort. (**C**) A nomogram was established to predict the prognosis of Kidney Renal Clear Cell Carcinoma (KIRC) patients. (**D**) Calibration plots showing the probability of 1-, 3-, and 5-year overall survival in TCGA cohort. (**E**) Kaplan-Meier analyses for the two KIRC groups based on the nomogram score. (**G**) Receiver operator characteristic (ROC) analysis of the nomogram in TCGA cohort

Subsequently, univariate and multivariate Cox regression analyses were conducted to determine if PCDs could act as an independent prognostic factor. Univariate Cox regression analysis showed that PCDs were considered a risk factor (HR = 3.53, 95% CI: 2.78–4.47, Fig. 6A). The results of a multivariate analysis, adjusted for other confounding factors, still designated PCDs as an independent prognostic factor for patients with KIRC (HR = 2.79, 95% CI: 2.15–3.63, Fig. 6B). A nomogram model was established using multivariate Cox and stepwise regression analyses to estimate the survival at 1, 3, and 5 years, incorporating age, stage, and PCDs (Fig. 6C). Calibration curves demonstrated the accuracy of this model in predicting the 1, 3, and 5-year survival rates (Fig. 6D). Significant survival differences across different subgroups were observed based on nomogram scores (Fig. 6E). The ROC analysis suggested the nomogram provided high accuracy in predicting 1-year, 3-year, and 5-year survival in KIRC patients (Fig. 6F).

### TOP2A is associated with proliferation and metastasis in KIRC cells

**Fig. 7:**
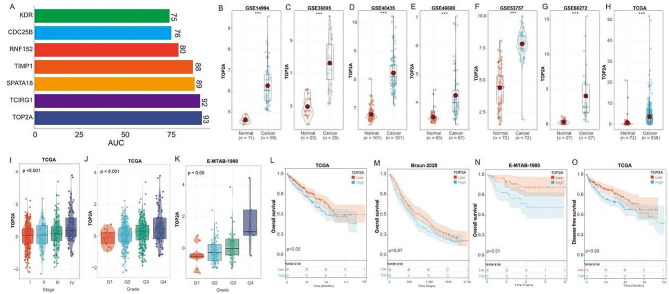
TOP2A is overexpressed in KIRC and associated with poor prognosis. (**A**) Area under the curve (AUC) analysis of seven genes distinguishing KIRC tissue from adjacent non-cancerous tissue; Expression of TOP2A in KIRC tissue and adjacent non-cancerous tissue in (**B**) GSE14994, (**C**) GSE36895, (**D**) GSE40435, (**E**) GSE46699, (**F**) GSE53757, (**G**) GSE66272, (**H**) TCGA cohort. (**I**) Expression analysis of the TOP2A gene at different stages in the TCGA cohort. (**J**) Expression analysis of the TOP2A gene in different grades in the TCGA cohort. (**K**) Expression analysis of the TOP2A gene in different grades in the E-MTAB-1980 cohort. OS analysis of TOP2A in (**L**) TCGA, (**M**) Braun-2020, and (**N**) E-MTAB-1980 cohorts. (**O**) Disease-free survival (DFS) analysis of TOP2A in TCGA cohort. ***, *p* < 0.001

Next, we assessed the diagnostic utility of these 7 PCD genes between KIRC tissues and adjacent non-tumor tissues, with ROC analysis showing that TOP2A had the highest AUC value (Fig. 7A). In the GSE14994, GSE36895, GSE40435, GSE46699, GSE53757, GSE66272, and TCGA cohorts, TOP2A expression was significantly elevated in KIRC compared to adjacent non-tumor tissues (Fig. 7B-H). TOP2A was associated with higher tumor grade and advanced pathological stage (Fig. 7I-K). High TOP2A expression was associated with worse overall survival (OS) and disease-free survival (DFS) in patients with KIRC (Fig. 7L-O).

**Fig. 8:**
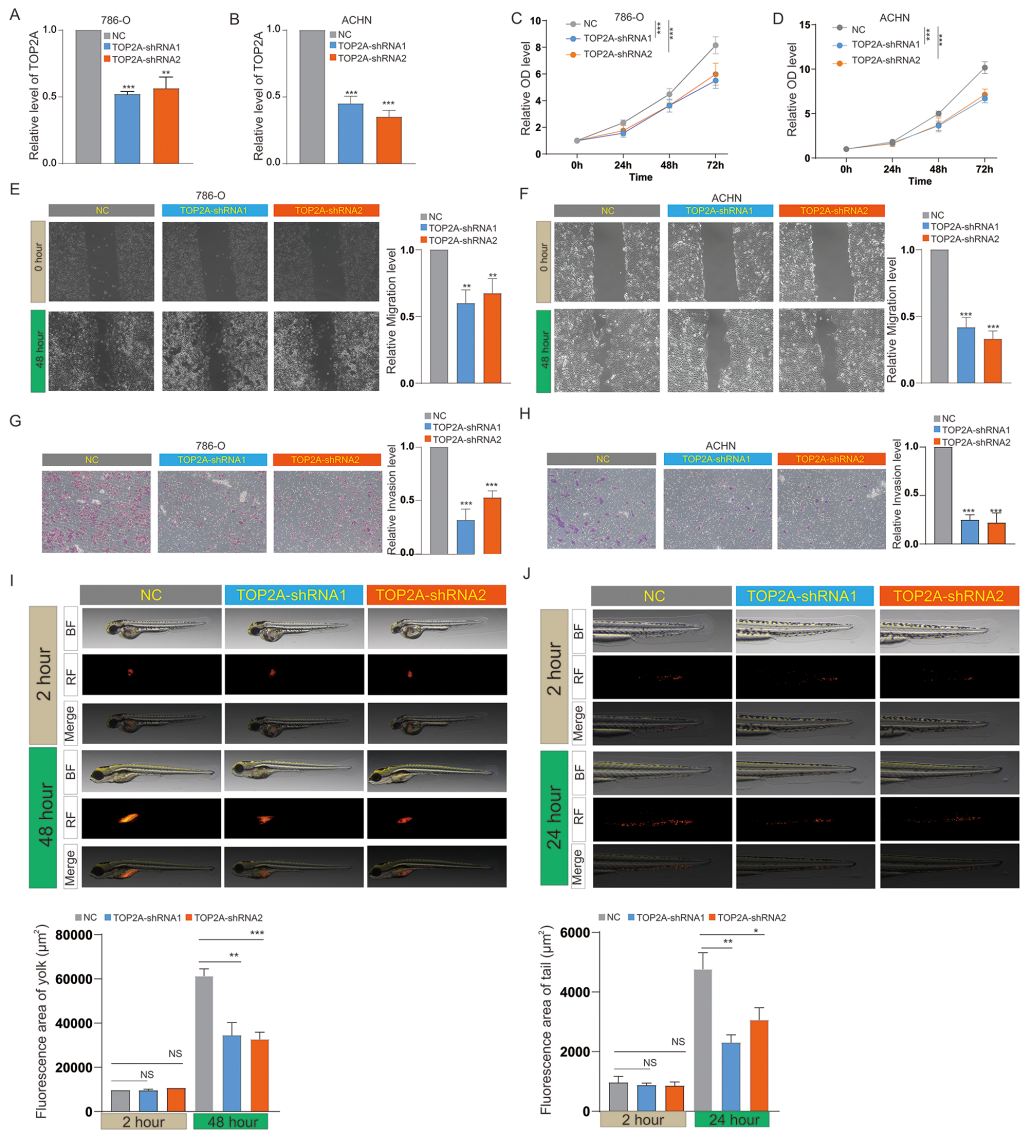
TOP2A promotes proliferation and metastasis of KIRC cells. RT-PCR detection of TOP2A expression in (**A**) 786-O and (**B**) ACHN cells after knockdown of TOP2A. CCK8 assay to measure changes in proliferation ability after knockdown of TOP2A in (**C**) 786-O and (**D**) ACHN cells. Effect of TOP2A knockdown on (**E**) migration and (**G**) invasion ability of 786-O cells. Effect of TOP2A knockdown on (**F**) migration and (**H**) invasion ability of ACHN cells. Impact of TO P2A knockdown on (**I**) proliferation and (**J**) metastasis of ACHN cells in zebrafish. ns, *p* > 0.05; *, *p* < 0.05; **, *p* < 0.01; ***, *p* < 0.001

To investigate the impact of TOP2A on the functional behavior of KIRC cells, we silenced TOP2A in 786-O and ACHN cells (Fig. 8A-B). Results from CCK8 assays indicated that knockdown of TOP2A significantly inhibited the proliferation of 786-O and ACHN cells (Fig. 8C-D). Wound healing and invasion assays demonstrated that TOP2A knockdown significantly repressed the migration and invasion abilities of 786-O and ACHN cells (Fig. 8E-H). Moreover, we further analyzed the effects of TOP2A knockdown on in vivo proliferation and metastatic capabilities of ACHN cells using a zebrafish model. The results suggested that knockdown of TOP2A significantly inhibited the in vivo proliferation and metastasis of ACHN cells (Fig. 8I & J).

## Discussion

The pivotal significance of Programmed Cell Death (PCD) in biological fundamentals has been steadily substantiated by mounting evidence [25, 26]. PCD has been confirmed to be associated with the incidence and metastasis of malignant tumors [27, 28]. Recent research reveals that PCD characteristics manifest a robust predictive capacity in triple-negative breast cancer, and hold potential for assessing tumor immune microenvironments and responsiveness to adjunct chemotherapy [8]. Two studies have already explored the relationship between PCD and KIRC. Our research shares some similarities with previous publications but also elucidates the unique contribution of our study.We utilized a comprehensive and rigorous approach in constructing our prognostic model by integrating three independent renal cancer datasets for univariate survival analysis, followed by an intersection for LASSO Cox regression analysis. This method contrasts with the studies by Zhang, Xi, et al. [29]., and Wu, Zhengqi, et al. [30]., where only TCGA data were leveraged, and in Zhang et al.‘s case, single-cell data intersection may have limited the gene selection. Our model’s superior accuracy is substantiated by the ROC curve AUC values, which outperform those reported in the aforementioned studies. These findings underscore the robustness of our prognostic mode.

Within our investigation, we designed a signature that comprises seven PCD genes (CDC25B, KDR, RNF152, SPATA18, TCIRG1, TIMP1, and TOP2A) and unveiled its predictive prowess regarding the survival rate of KIRC patients. CDC25B is a critical member of the CDC25 gene family, playing an indispensable role in cell cycle progression. Recent research has showcased an overexpression of the CDC25B gene in a spectrum of human cancers, including breast cancer, nasopharyngeal carcinoma, and hepatocellular carcinoma. This overexpression has been linked to poorer survival rates and is implicated in the progression of KIRC [31]. KDR, a pivotal receptor in both vasculogenesis and angiogenesis, processes that are crucial for tumor growth and progression, has been found to exhibit increased expression that correlates with a poor prognosis in renal cell carcinoma [32]. RNF152 is part of the RNF protein family, is known to undergo polyubiquitination via its RING finger domain. It has been reported to inhibit the growth of colorectal cancer cells [33]. The SPATA18 gene codes for a protein with the ability to induce lysosome-like organelles within mitochondria. Notably, a higher expression rate of SPATA18 in KIRC has been linked to a more favorable prognosis [34]. TCIRG1, a gene crucial for cellular life functions through its acidification dependency, acts as a promoter for tumor growth by influencing aerobic glycolysis and the tumor immune microenvironment in KIRC [35]. TIMP1, or Tissue Inhibitor of Metalloproteinases 1, is an inhibitor of the matrix metalloproteinases. Interestingly, TIMP1 contributes to an immunosuppressive microenvironment by regulating anoikis, thereby propping up the progression of KIRC [36]. TOP2A acts as a key catalytic enzyme triggering DNA replication. Significant overexpression of the TOP2A gene has been noted across a range of human cancers [37].

Indications suggest that tumors classified under the High-PCDs subtype exhibit a more aggressive biological demeanor, owing to their pronounced association with higher tumor grades and advanced stages of pathology. Additionally, tumors in the High-PCDs subtype have an immune suppressive phenotype, with an infiltration of more Tregs and tumor-associated macrophages (TAM), accompanied by an overexpression of genes that negatively regulate the anti-tumor immune process. Concurrently, we discovered that tumors under the Low-PCDs subtype have an enhanced response to anti-PD-1 drugs.

Tumor cells are permitted to thrive as the tumor microenvironment provides sanctuary against immune detection and drug intervention. Our research unveils the relationship between PCDs and immune microenvironment. Both Tregs and TAMs are more infiltrated in High-PCDs subtype tumors. Tregs, uniquely characterized by FOXP3 expression [38], can suppress immune activation through the secretion of immune repressive cytokines or expression of co-suppressive molecules. Whereas the TAMs, exemplifying M2 polarization, represent the most populous subgroup of white blood cells within cancer, molded by chemokines that impede anti-tumor immunity [39–41]. Employing both CIBERSORT and XCELL for our analysis revealed heightened levels of Tregs and TAMs under the High-PCDs subtype. These findings pave the way towards a refined understanding of PCD patterns’ influence on immune responses, and in turn, may greatly aid the creation of more effective immunotherapy methods, such as combining PCD-targeting drugs with immune-checkpoint inhibitors.

Despite the global scale of immunotherapeutic clinical trials currently in progress, propelling the usage of ICIs in KIRC, the remission rate (5-12.5% for CTLA-4 inhibitors and 20–27% for PD-1 inhibitors) remains relatively low compared to other solid tumors. This is despite prior studies indicating benefits to KIRC patients upon inhibition of immune checkpoints like CTLA-4 and PD-1/PD-L1 [2].

The role of PD-L1 as a biomarker for predicting the ICI response in major malignant tumors, including KIRC, is a disputed topic. The outcome of the CHEKMATE-025 trial of treating refractory mRCC with Nivolumab and Everolimus showed consistent improvement in overall survival regardless of PD-L1 expression [42]. Our research suggests that high PCD KIRC patients exhibit unfavorable survival outcomes post-immunotherapy. As such, this implies that PCD-high patients potentially represent a segment intolerant to PD-1 blockade therapy. This revelation has been reinforced in melanoma cohorts, suggesting that PCDs might have general predictive abilities in determining immune therapeutic responses across a multitude of malignant tumors. Therefore, stratifying patients based on PCD identifiers could enhance personalized immunotherapy methods and increase beneficial outcomes. Given these conclusions, we proceeded to construct a nomogram employing PCDs as a proficient prognostic tool for predicting the potential prognosis of KIRC.

TOP2A plays a crucial role in the processes of DNA replication, transcription, recombination, and repair by altering the topological states of DNA. Specifically, TOP2A works by creating transient double-stranded breaks in the DNA molecule, which allows the manipulation of DNA topology, including relaxing supercoiled DNA, untangling interlinked DNA strands, and facilitating chromosome segregation during cell division. In cancer, the role of TOP2A is of particular interest because of its involvement in the proliferation of cells. Given that cancer is characterized by uncontrolled cell growth and division, enzymes like TOP2A that are essential for DNA replication are often found to be overexpressed in various tumor types. Overexpression of TOP2A has been linked to a more aggressive tumor phenotype and is associated with poor prognosis. Based on these research insights, TOP2A emerges as a potential biomarker for the screening, diagnosis, prognosis, and monitoring of these types of tumors. In our study, we discovered a noteworthy overexpression of TOP2A in KIRC, which correlated with poorer prognosis. Moreover, the suppression of TOP2A significantly hindered the growth and metastasis of KIRC cells, both in vivo and in vitro. Thus, TOP2A holds significant promise as a molecular target for groundbreaking preventative and therapeutic strategies in KIRC patients. However, it is crucial for future studies to thoroughly decode the molecular mechanisms behind TOP2A dysregulation and its consequential role in KIRC progression.

Despite the comprehensive nature of our research, we acknowledge several limitations. First, our findings are based on retrospective data, which may introduce potential bias. Future well-designed propective trials are needed to confirm these findings. Second, our research primarily relied on transcriptome sequencing data and corresponding clinical information from public databases. Hence, the findings may not fully capture the dynamics and complexity of immune interactions and tumor microenvironment given the inherent limitations of such data sources. Our future research will focus on overcoming these limitations to further enhance our understanding of the complex interplay between PCD, tumor immune microenvironment, and immunotherapy responses.

## Conclusion

In conclusion, our study represents an initiative to elucidate the complex relationship between PCD gene characteristics and KIRC prognosis. We devised a unique PCD gene signature composed of seven genes that exhibited significant predictive capability for KIRC patient’s survival. Our findings also highlighted that the tumor microenvironment is considerably altered in high-PCDs phenotype owing to an excessive infiltration of immune suppressive cells such as Tregs and tumor-associated macrophages. This leads to an immune evasion phenomenon thereby causing a poor response to ICI therapy in high-PCDs patients.

### Electronic supplementary material

Below is the link to the electronic supplementary material.

Supplementary Material 1

Supplementary Material 2

Supplementary Material 3

Supplementary Material 4

Supplementary Material 5

## Data Availability

The datasets used to support the conclusion of this study were collected from publicly available databases including Cancer Genome Atlas database (https://portal.gdc.cancer.gov/) and Gene Expression Omnibus database (https://www.ncbi.nlm.nih.gov/geo/). In order to ensure the reproducibility of the experimental results, we have uploaded all the source code used in this study to the Figshare online data storage platform. The code can be accessed via the following link: https://figshare.com/s/5036bc342f2411af84e6.
