# Supplementary material for: Assessing the role of programmed cell death signatures and related gene TOP2A in progression and prognostic prediction of clear cell renal cell carcinoma
Source: Cancer Cell Int. 2024 May 10;24:164. doi: 10.1186/s12935-024-03346-w (PMC11084013; doi:10.1186/s12935-024-03346-w)

Supplementary Figure 1

ROC curve analysis


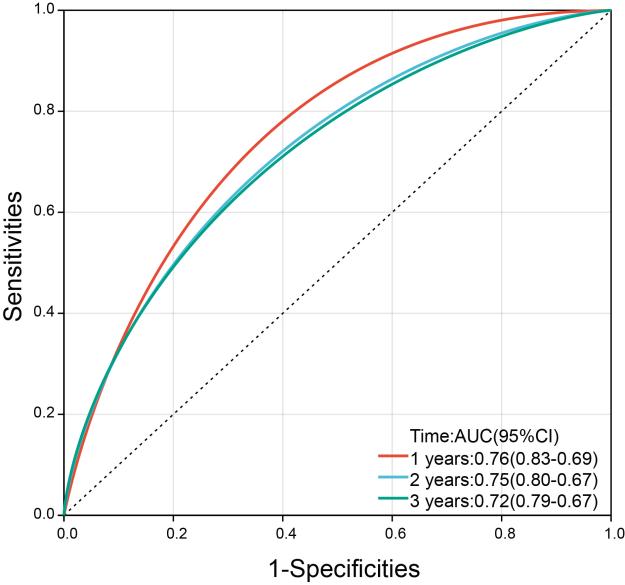


Supplementary Figure 2

Unsupervised clustering of PCD related model genes. When k=2, KIRC patients were grouped into two molecular clusters based on the PCD model gene profile in the (A) TCGA, (E) E-MTAB-1980, and (I) Braun-2020 cohorts. The empirical cumulative distribution function plots display consensus distributions for each k value in the (B) TCGA, (F) E-MTAB-1980, and (J) Braun-2020 cohorts. Kaplan-Meier analysis of the prognosis of KIRC patients belonging to two different molecular clusters in the (C) TCGA, (G) E-MTAB-1980, and (K) Braun-2020 cohorts. The alluvial diagram shows the interrelationship between molecular clusters, survival status, and CDKs groups in KIRC patients in the (D) TCGA, (H) E-MTAB-1980, and (L) Braun-2020 cohorts.


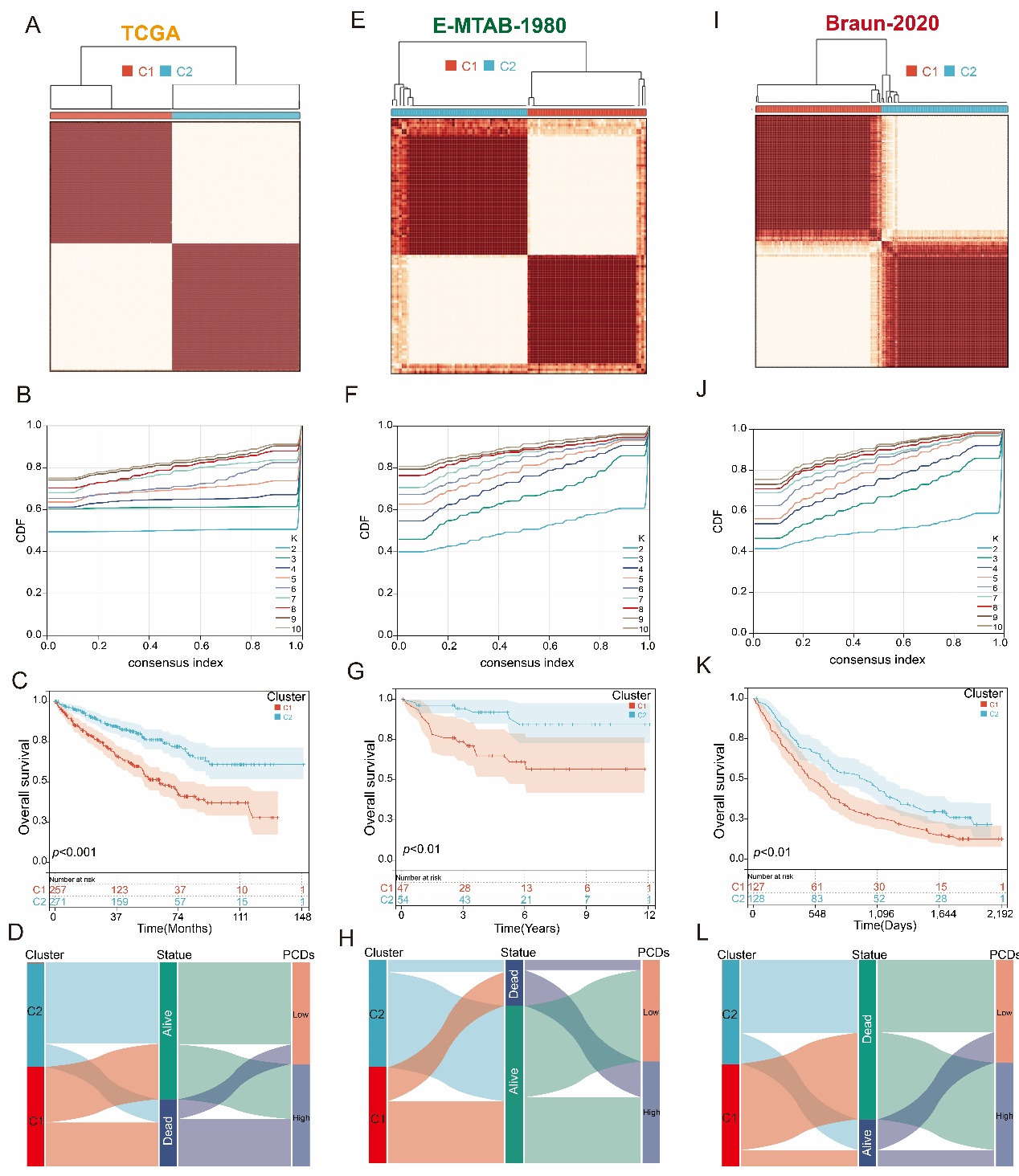

Supplement: Supplementary file 5 — Supplementary Material 5 [file 12935_2024_3346_MOESM5_ESM.docx]
